# A Self-Healing, Transparent, and Hydrophobic Flame-Retardant Coating for Wood Based on Bio-Derived Flame Retardants and Fluorosilane Surface Treatment

**DOI:** 10.3390/polym18121497

**Published:** 2026-06-15

**Authors:** Lu Liu, Hongfei He, Xiaming Feng, Ming Fu, Hongyu Yang, Bin Yu

**Affiliations:** 1 College of Materials Science and Engineering, Chongqing University, 174 Shazhengjie, Shapingba, Chongqing 400044, China; 2 State Key Laboratory of Fire Science, University of Science and Technology of China, Hefei 230026, China; 3 State Key Laboratory of Safety and Resilience of Civil Engineering in Mountain Area, Chongqing University, Chongqing 400044, China; 4 Hefei Institute for Public Safety Research, Tsinghua University, Hefei 230601, China

**Keywords:** wood coating, flame retardancy, gelatin, transmittance, bio-based protective coatings

## Abstract

Wood’s inherent flammability, arising from its cellular organic composition, demands effective protective strategies. This study aimed to develop a multifunctional bio-based wood coating simultaneously integrating flame retardancy, optical transparency, moisture-triggered self-healing, and surface hydrophobicity within a single formulation. An intumescent flame retardant (PAGHR) was synthesized via ionic assembly of a phytic acid–phosphorylated polyethylene glycol conjugate (PgP) with a piperazine–etidronic acid salt (HEPHR), subsequently blended with gelatin (G) and surface-finished with fluorosilane. The optimized coating (G/PAGHR-4) achieved a limiting oxygen index (LOI) of 37.2% and passed the UL-94 V-0 rating. Cone calorimetry demonstrated reductions of 75.1% in peak heat release rate (pHRR) and 50.0% in total heat release (THR) relative to the neat gelatin control. Char yield at 700 °C increased substantially from 17.8 wt% to 41.0 wt%, confirming effective condensed-phase char promotion. Beyond fire performance, the coating maintained high visible-light transmittance, preserved natural wood aesthetics, and achieved macroscopic scratch healing within 40 min upon ambient water contact. Fluorosilane finishing elevated the water contact angle to 122°. These results establish a scalable, environmentally friendly strategy for multifunctional bio-based protective coatings applicable to wood, textiles, and polymer substrates.

## 1. Introduction

Wood is among the most widely used renewable structural and decorative materials. Its light weight, workability, and natural aesthetic appeal make it a preferred choice in building interiors, furniture, and transportation fittings [1,2,3,4,5]. However, the same organic cellular composition that confers these advantages also renders wood readily combustible [6]. Upon ignition, rapid pyrolysis generates a continuous supply of flammable volatiles that sustain and intensify flame spread, while heat release escalates quickly during fire exposure [7,8,9,10]. Improving the fire safety of wood without compromising its appearance or structural function therefore remains a problem of genuine practical importance [11,12,13].

Surface flame-retardant coatings are among the most attractive protection strategies, as they leave bulk geometry and mechanical properties intact. They can be applied by brush, roller, or spray onto irregular shapes, and their composition can be adjusted for specific fire scenarios [14,15,16,17]. Intumescent formulations are of particular interest. Under heat, they swell into an expanded carbonaceous foam that retards conductive heat transfer, limits oxygen supply to the combustion front, and suppresses the outward flux of combustible pyrolysis gases [5,18,19]. A persistent limitation of many conventional intumescent products, however, is their dependence on petroleum-derived components. They also tend to form opaque, moisture-sensitive films that are incompatible with applications requiring a clear, natural wood finish [20,21,22]. Transparent coatings that preserve the grain character of the substrate, combined with moisture-stable films that resist leaching of active flame-retardant species, are therefore both practically desirable and technically demanding targets [23].

Bio-derived flame-retardant chemistry has attracted sustained research interest as a halogen-free and renewable alternative to conventional petroleum-based systems [9,24,25,26,27,28,29,30]. Phytic acid (PA), the hexaphosphoric ester of myo-inositol found abundantly in plant seeds, is notable for its high phosphorus content and strong char-catalysing capacity [22,31,32]. Liu et al. demonstrated that PA-derived polyphosphate networks can function simultaneously as an acid source, carbon source, and cross-linking agent within a single bio-based building block, yielding efficient intumescent char formation on both wood and polymer substrates [26]. Nevertheless, direct use of unmodified PA as a coating component is constrained by its strong acidity, poor film-forming tendency, and marked sensitivity to moisture leaching [1,33,34]. Modification strategies that retain phosphorus density while improving processability are therefore required. Combining PA with structurally complementary partners through covalent modification and ionic co-assembly offers a practical route to address these constraints [35,36]. Etidronic acid (HEDP), a geminal bisphosphonate, introduces thermally robust P–C linkages alongside the phosphate ester bonds of PA. Piperazine provides nitrogen-bearing units that participate in gas-phase radical quenching and intumescent blowing. Gelatin, a naturally derived protein rich in amino, amide, and hydroxyl side chains, serves as a film-forming matrix with abundant reversible non-covalent interaction sites [27,37,38].

Beyond flame retardancy, functional wood coatings must satisfy several additional requirements in real service environments [39,40]. Optical transparency is necessary wherever the natural wood grain must be preserved. Self-healing capacity extends coating service life by restoring surface continuity after mechanical damage [41]. Surface hydrophobicity counteracts the moisture-leaching vulnerability common to phosphorus–nitrogen intumescent systems [24,42,43,44]. Satisfying all four attributes—flame retardancy, transparency, self-healing, and hydrophobicity—within a single coherent coating formulation remains a significant challenge [24,43]. Each property imposes distinct structural demands at the molecular level. These demands are not fully compatible and partially conflict with one another. Achieving a balanced design therefore requires careful consideration of molecular architecture [42,45,46].

Several recent studies have addressed subsets of these requirements. Sun et al. developed a flame-retardant wood coating with integrated anti-wetting, self-cleaning, and good weatherability properties; however, ambient-condition self-healing was not incorporated [47]. Liang et al. reported a bio-based vanillin-derived epoxy coating crosslinked with amino-functionalised POSS (VE/OA-POSS) that achieved a UL-94 V-0 classification with an LOI of 33.1% and exceptional mechanical properties (pencil hardness 6H). The flame-retardant performance was attributed to a silicon-doped condensed-phase char barrier coupled with gas-phase dilution by non-combustible gases. Nevertheless, self-healing in that system required thermal activation at 65 °C, and autonomous healing under ambient conditions was not realized [48]. These prior efforts collectively highlight that no single bio-derived coating system has yet reconciled all four functional attributes within one chemically unified architecture.

**Figure 1:**
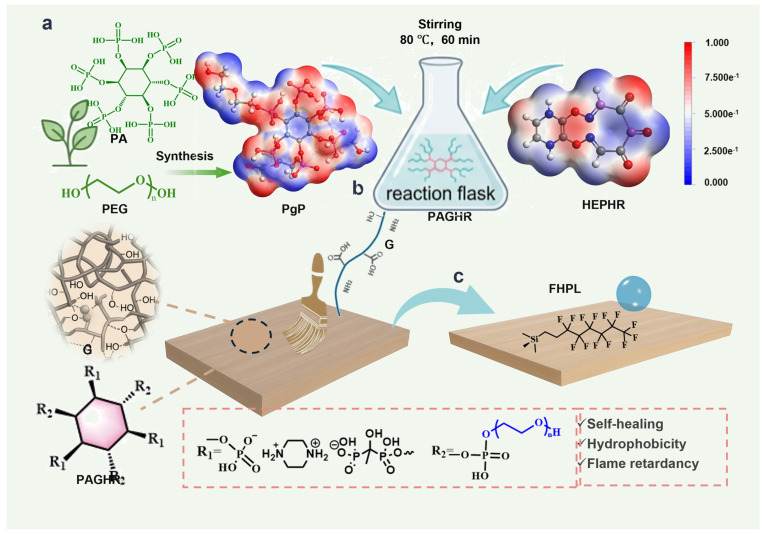
(**a**) The synthetic scheme of PgP and PAGHR. (**b**) Schematic diagram of a transparent G/PAGHR as a highly efficient flame-retardant composite coating on wood. (**c**) Schematic diagram of hydrophobic treatment for flame-retardant coating.

In the present work, phytic acid (high phosphorus content, natural origin), HEDP (introduction of thermally stable P–C bonds), piperazine (gas-phase radical quenching and char-foaming functionality), and gelatin (natural film-forming matrix rich in dynamic hydrogen-bonding crosslinking sites) were selected based on their complementary functional roles. PgP and HEPHR were assembled ionically and blended with gelatin to produce the G/PAGHR coating series (Figure 1a and Figures S1–S3). The hybrid coating was deposited as a transparent film on wood (Figure 1b), with optional FHPL fluorosilane finishing to impart hydrophobic durability (Figure 1c). To achieve simultaneous flame retardancy, optical transparency, moisture-triggered self-healing, and surface hydrophobicity within a single bio-based coating system. This work represents the first integration of phytic acid–PEG phosphate ester and piperazine–HEDP salt via ionic assembly into a gelatin matrix, constructing a unified molecular architecture that fulfills all four functional requirements simultaneously, which is clearly distinguished from existing systems that address only a subset of these properties [43,48].

## 2. Results and Discussion

### 2.1. Fabrication of Coating and Structural Characterizations

#### 2.1.1. DFT Analysis of PgP Configuration and ELF Analysis

The structural preference of the phosphorylated phytic acid–PEG derivative was first analyzed by DFT calculations. As shown in Figure S4 and Table S2, three possible substitution patterns of PEG grafting onto phytic acid, namely (1,2,3), (1,3,4), and (1,3,5), were considered. Among these three possibilities, the (1,3,5) configuration gives the lowest total energy (−17,932.44203 eV), which is lower than those of the (1,2,3) and (1,3,4) configurations. From a structural chemistry perspective, the preferential stability of the (1,3,5) configuration is further supported by the molecular geometry revealed in Figure S4c: the molecule adopts a relatively extended, planar-like conformation with the PEG chains splayed outward symmetrically, suggesting reduced intramolecular steric and electrostatic repulsion between the phosphate ester linkages and the pendant PEG segments. The thermodynamic preference for the (1,3,5) configuration implies that, under the thermal reaction conditions employed, the esterification of PEG onto the inositol phosphate framework is stereo-electronically guided toward the energetically favored alternating substitution pattern. This result indicates that the (1,3,5) arrangement is energetically preferred and likely represents the most stable substitution mode for PgP.

ELF maps for PgP and HEPHR (Figure S5) were computed to visualise local bonding character in the two building blocks. High localisation (ELF → 1.0) around oxygen atoms of the phosphate and phosphonate units reflects the strongly polar nature of these groups in both structures. Within the PgP map, well-defined ELF domains at C–O esterification linkages confirm covalent bond formation at the grafting points; the absence of compressed or overlapping lobes between adjacent PEG arms is consistent with the steric compatibility of the (1,3,5) arrangement identified above. For HEPHR, the coexistence of phosphonate groups and protonated nitrogen-containing units suggests the presence of chemically distinct regions with different bond polarities. It can be observed that a proton transfers from the P–OH group to the N atom, forming an N–H bond. Furthermore, the ELF map in Figure S5d reveals a distinct N–H–O interaction: the electron density of the N–H bond is localized, whereas that in the H–O region is delocalized. This indicates that electrons are enriched on the N–H side and depleted on the O–H side, giving rise to polarity and exhibiting characteristics of an ionic bond. Although ELF analysis does not directly determine decomposition temperature, it supports the interpretation that the phosphorus-containing and nitrogen-containing segments may respond differently during heating, which is consistent with the staged degradation behavior observed in the thermal analysis. Proton Transfer and N–H Bond Formation. Upon geometric relaxation, spontaneous proton transfer from a P–OH group of HEDP to the terminal nitrogen atom of piperazine is observed, resulting in the formation of a covalent N–H bond at the piperazinium center. This is consistent with the acid–base ionic complexation mechanism underlying HEPHR synthesis and confirms that the protonation state depicted in the structural formula is energetically favorable in the gas-phase optimized geometry.

Taken together, the DFT and ELF results provide a theoretical basis for the structural organization of the hybrid P–N system and support the subsequent discussion of its thermal decomposition behavior [49,50].

#### 2.1.2. ^1^H and ^31^P NMR Analysis

The structures of HEPHR and PAGHR were examined by ^1^H NMR spectroscopy (Figure S6a). In the spectrum of HEPHR, the resonances appearing in the 2.4–3.6 ppm range can be assigned to the methylene protons of the piperazinium ring (–NCH_2_CH_2_N–) together with the methyl protons associated with the HEDP-derived geminal bisphosphonate unit [–C(CH_3_)(PO_3_H_2_)_2_]. These features are consistent with the expected salt formation through acid–base interaction between HEDP and piperazine [51]. Upon going to PAGHR, the same environments are identifiable but with noticeably broadened line shapes—a consequence of the more constrained segmental dynamics in the multi-component ionic network, where contacts among PgP, HEPHR, and gelatin functional groups restrict local chain motion.

Additional information on the phosphorus environments was obtained from ^31^P NMR spectroscopy (Figure S6b). HEPHR shows a broad resonance centered near 18 ppm, which is characteristic of the phosphonate environment in HEDP-derived structures. The broad line shape suggests a distribution of local chemical environments, which is reasonable for an ionically bonded salt. By contrast, PAGHR displays a dominant sharp resonance in the 0–1 ppm region, indicating a distinct phosphorus environment after assembly. This signal is mainly attributed to phosphate ester species in the PgP component, especially P–O–C environments formed between phytic acid and PEG. Liu et al. similarly reported that phytic acid-derived polyphosphate assemblies display characteristic upfield ^31^P resonances attributable to phosphate monoester coordination environments [26].

#### 2.1.3. Moisture-Mediated Self-Healing

**Figure 2:**
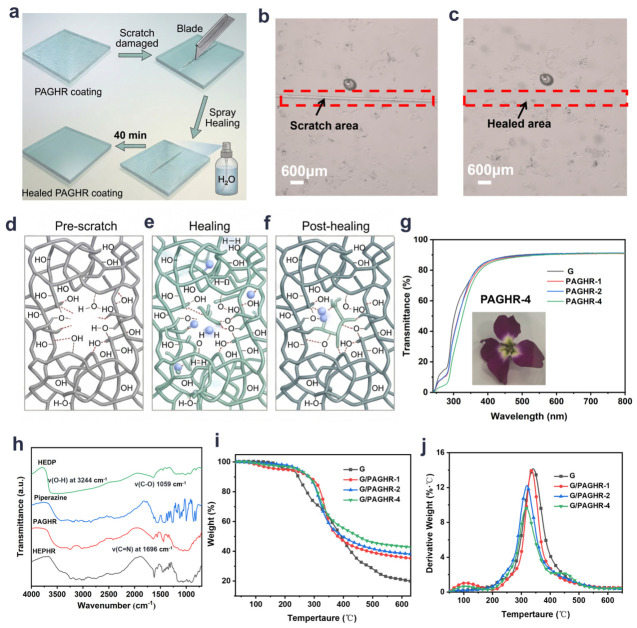
(**a**) Moisture-mediated self-healing of the G/PAGHR-4 coating. The scratch for assessing self-healing properties. Scratch-defected optical microscopic (OM) images for before (**b**) and after healing (**c**). (**d**–**f**) Schematic illustrations showing self-healing mechanisms occurring in self-healing coatings. (**g**) The curves of transmittance spectra of the G and G/PAGHR coatings. (**h**) FTIR spectra of HEDP, P, PAGHR and HEPHR. (**i**) TGA and (**j**) DTG curves of the G and PAGHR coatings under N_2_ atmosphere.

Scratch-healing experiments on G/PAGHR-4 are illustrated in Figure 2a–c. After a scratch is introduced, a visibly damaged region appears on the coating surface. Following water spraying and standing at room temperature for 40 min, the scratch becomes much less apparent, indicating effective healing of the damaged area. The schematic diagrams in Figure 2d–f further suggest that this recovery process is associated with dynamic supramolecular reconstruction in the coating. Upon mechanical scratching, the hydrogen bond network at the damage zone is disrupted (Figure 2d). When water is introduced via spray application (Figure 2e), water molecules diffuse into the scratch channel. Based on the well-documented plasticizing effect of water on gelatin-based matrices and hydrogen-bond-rich polymer networks [52,53], increased segmental mobility in the water-saturated damaged zone is expected to facilitate rearrangement of the disrupted hydrogen-bonding network. Upon subsequent evaporation of water, the reconstituted non-covalent interactions restore coating continuity at the scratch site. Simultaneously, the water molecules mediate the reformation of hydrogen bonds by bridging the fractured network segments through O–H···O and N–H···O interactions involving the piperazinium N–H groups of HEPHR and the phosphate/hydroxyl oxygen acceptors of PAGHR. After evaporation, the reconstituted hydrogen-bond network locks in the healed configuration. After water is introduced, it diffuses into the damaged region and acts as a temporary plasticizing medium, which increases the mobility of the polymer chains and ionic assemblies in the scratched area. At the same time, water facilitates the rearrangement of hydrogen-bonding interactions among the functional groups in the network. As the water gradually evaporates, the reorganized hydrogen-bond network becomes re-established, leading to recovery of the coating continuity at the damaged site.

Therefore, the self-healing behavior of G/PAGHR-4 can be understood as a water-assisted reconstruction of the dynamic hydrogen-bonding network. This process does not require external heating or additional healing agents, which is advantageous for practical surface coating applications. The introduction of water molecules may lower the glass transition temperature of the coating through a plasticizing effect, thereby enhancing segmental mobility and facilitating scratch closure—a behavior consistent with the reported moisture-induced plasticization of gelatin matrices. Direct verification by DMA or rheological measurement is planned for future work.

#### 2.1.4. Optical Transparency

The optical transparency of the G and G/PAGHR coatings was evaluated in the wavelength range of 300–800 nm. As shown in Figure 2g, all samples maintain relatively high transmittance in the visible region (400–800 nm), indicating that the introduction of PAGHR does not significantly impair the light transmission of the coating. In the inset photograph, the G/PAGHR-4-coated substrate remains clearly visible, further confirming the transparency of the coating at the macroscopic level. At 550 nm, the transmittance of G and G/PAGHR-4 is 90.62% and 89.82%, respectively, differing by only 0.8% (Figure 2g and Figure S10). The good optical performance of the G/PAGHR system can be attributed to the homogeneous nature of the coating matrix. As discussed in the morphology analysis, the elemental mapping results indicate a uniform distribution of the constituent elements throughout the coating, suggesting the absence of large aggregates or obvious phase-separated domains that could lead to strong light scattering. In addition, the PEG segments in the PgP component are beneficial for the miscibility and film-forming behavior of the aqueous system, which helps maintain the optical uniformity of the dried coating.

These results show that the G/PAGHR coating can provide flame-retardant functionality while preserving the visual appearance of the wood substrate. This feature is particularly important for wood protection applications in which the natural texture and decorative value of the substrate need to be retained [42].

#### 2.1.5. FTIR Analysis

The formation of HEFER and PAGHR was further examined by FTIR spectroscopy. In the spectrum of HEDP, the broad absorption band at 3244 cm^−1^ is assigned to the stretching vibration of phosphonic acid hydroxyl groups, while the absorption at 1059 cm^−1^ is associated with the phosphorus-containing framework. After reaction with piperazine, the spectrum of HEFER shows a new band at 1696 cm^−1^, which can be assigned to the bending vibration of protonated N–H species. This feature is absent in the spectra of neat HEDP and piperazine, indicating that proton transfer occurs during salt formation and supporting the formation of the HEFER ionic pair.

The FTIR spectrum of PAGHR retains the main absorption features associated with both the PgP and HEFER components, with slight shifts in several bands. These changes are consistent with the presence of hydrogen-bonding and electrostatic interactions in the assembled network. Combined with the ^1^H and ^31^P NMR results, the FTIR data support the proposed structure of HEFER as a piperazinium–HEDP ionic salt and PAGHR as a multicomponent ionic assembly containing both phosphate- and phosphonate-based phosphorus species. This structural organization is expected to influence the subsequent thermal decomposition behavior and, consequently, the flame-retardant action of the coating system.

### 2.2. Thermal Degradation Behavior of G/PAGHR Coatings

#### TGA and DTG Analysis

Thermogravimetric data acquired under nitrogen for G and the G/PAGHR series are collected in Figure 2i,j and Figure S18a and Table S4. The thermal decomposition behavior of the neat glutaric acid-based cross-linker (G) and the G/PAGHR composite coatings at three loading levels (G/PAGHR-1, G/PAGHR-2, and G/PAGHR-4) was evaluated by TGA under nitrogen atmosphere. Neat G undergoes rapid and near-complete decomposition in the temperature range of approximately 200–350 °C, with a residual char yield approaching zero at 600 °C, reflecting the inherently poor char-forming capacity of the organic cross-linker alone. In contrast, all G/PAGHR composite coatings exhibit markedly enhanced thermal stability and char retention relative to neat G. Neat G loses 5% of its mass at T5% = 219 °C, reaches maximum decomposition rate at Tmax = 340 °C, and retains only 17.8 wt% char at 700 °C. The modest residue reflects the limited intrinsic carbonisation capacity of gelatin alone. The addition of PAGHR consistently and substantially raised the char yield across the entire composition series. G/PAGHR-1 already delivered a char residue of 33.3 wt%, although T5% dropped to 195 °C relative to neat G. This earlier onset of mass loss is attributed to thermally labile nitrogen-bearing linkages in HEPHR, which begin to cleave near 290 °C. G/PAGHR-2 recorded the highest T5% in the series at 257 °C, accompanied by a char yield of 36.8 wt%. G/PAGHR-4 exhibited a T5% of 244 °C, the lowest T_max_ in the series at 317 °C, and the highest char yield of 41.0 wt%. The progressive reduction in T_max_ reflects the growing contribution of PAGHR decomposition pathways to the overall mass-loss profile. Neat PAGHR loses 5% of its mass at T5% = 264 °C, reaches maximum decomposition rate at T_max_ = 288 °C, and retains 41.4 wt% char at 700 °C. With increasing PAGHR loading from G/PAGHR-1 to G/PAGHR-4, a progressive increase in the residual mass at 600 °C is observed, demonstrating a clear loading-dependent enhancement of char formation. This trend is consistent with the higher phosphorus and nitrogen content introduced by PAGHR, which promotes the cross-linking and condensation of carbonaceous residues through phosphate-catalyzed dehydration and P–N synergistic char-stabilization mechanisms. Meanwhile, the monotonic increase in char yield quantifies the phosphorus-catalysed conversion of organic fragments into stable carbonaceous residue, rather than volatile fuel. Comparable char yield enhancements have been reported for phytic acid (PA)-derived polyphosphate coatings on wood by Liu et al., who attributed the improvements to acid-catalysed substrate dehydration and condensed-phase cross-linking [2,26].

In contrast, all G/PAGHR coatings show broader and more complex DTG profiles, indicating that the introduction of PAGHR changes the degradation process from a single dominant step to a multi-stage thermal event. A shoulder peak appears at around 290 °C in the PAGHR-containing samples and becomes more evident with increasing PAGHR content. This early-stage mass-loss feature can be assigned to the decomposition of thermally less stable nitrogen-containing moieties in HEFER. Based on the ELF analysis, the relatively weak N–C bonds are expected to cleave preferentially, which is consistent with the appearance of this lower-temperature decomposition stage. This process is likely associated with the release of nitrogen-containing volatile species, which may contribute to gas-phase flame inhibition. At intermediate temperatures (approximately 320–336 °C), the major decomposition stage of the G/PAGHR coatings is observed. This stage likely includes overlapping degradation events from several components, including decomposition of phosphate-containing structures in PgP, degradation of PEG segments, and partial breakdown of the gelatin network. The formation of phosphorus-containing acidic species during this stage is expected to promote dehydration and carbonization of the matrix, thereby enhancing the formation of protective char. In addition, the G/PAGHR samples display a broader high-temperature tail extending above 400 °C, which is not obvious in neat G. This feature indicates the presence of thermally more stable phosphorus-containing structures that continue to participate in the degradation and carbonization process at an elevated temperature.

Therefore, the DTG results support a staged decomposition process in the G/PAGHR coatings. The early decomposition of nitrogen-containing structures, followed by phosphorus-promoted carbonization and the continued contribution of thermally stable phosphonate units at higher temperature, is consistent with the synergistic flame-retardant action of the hybrid P–N system. More importantly, this behavior agrees well with the bond-dependent thermal response suggested by the ELF analysis and helps explain the improved char yield and reduced fire hazard observed for the PAGHR-containing coatings.

### 2.3. Morphology and Coating Structure

#### 2.3.1. Surface Hydrophobicity and Hydrophobic Properties

**Figure 3:**
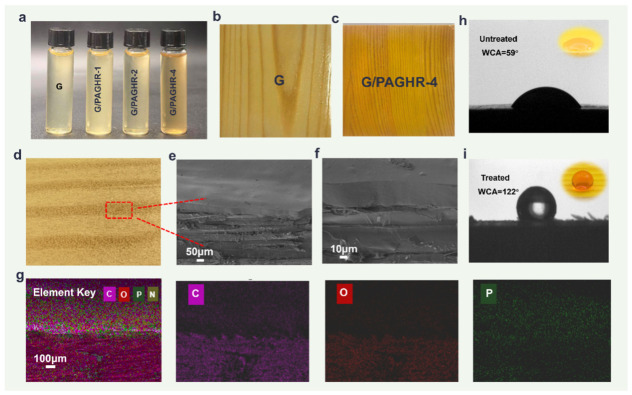
(**a**) Photographs of the corresponding dispersions. (**b**,**c**) Surface morphologies of wood samples treated. (**d**–**f**) The surface and cross-section SEM images of G/PAGHR-4 coating, and (**g**) corresponding EDS mapping images of C, O and P. The water contact angle of the untreated wood (**h**) and the treated wood (**i**).

This structural uniformity is also consistent with the good optical appearance of the dispersion system shown in Figure 3a. Moreover, the treated wood surfaces shown in Figure 3b,c and Figure S11a,b indicate that the coatings can cover the wood uniformly while maintaining visible wood texture, which is advantageous for decorative wood protection. The morphology of the G/PAGHR-4 coating was examined by SEM. As shown in Figure 3d–f, the coating forms a continuous and relatively smooth layer on the wood surface. The cross-sectional images further reveal that the coating is closely attached to the substrate, without obvious delamination or large internal voids. Such a continuous and dense structure is favorable for preserving transparency and for constructing an effective physical barrier during combustion. EDS elemental mapping shows that C, O, and P are uniformly distributed across the coating layer (Figure 3g), and the corresponding N mapping is presented in Figure S7. The homogeneous elemental distribution indicates that the phosphorus–nitrogen flame-retardant species are well dispersed within the coating matrix rather than aggregated in isolated domains.

#### 2.3.2. Surface Hydrophobicity

As shown in Figure 3h, static water contact angle measurements placed uncoated wood at WCA = 59°, consistent with the hydroxyl-rich, inherently hydrophilic character of the cellulose–lignin matrix. Following application of the G/PAGHR-4 coating and subsequent treatment with the fluorinated trichlorosilane coupling agent FHPL, the WCA increases markedly to 122° (Figure 3i), representing an increase of 63° relative to the untreated substrate and crossing the conventional hydrophobic threshold (WCA > 90°). This transition from hydrophilic to hydrophobic surface behavior is attributed to the covalent grafting of FHPL onto the coating surface: the trichlorosilane headgroup undergoes hydrolysis and condensation with surface hydroxyl groups present on the G/PAGHR coating, forming stable Si–O–substrate linkages, while the perfluoroalkyl tail chain orients away from the surface to present a densely packed, low-surface-energy fluorocarbon exterior.

The achievement of a WCA of 122° is of direct practical relevance to the durability of the flame-retardant coating. Phosphorus- and nitrogen-rich intumescent coatings based on ionic and hydrogen-bonded assemblies are inherently susceptible to moisture-induced leaching of the active flame-retardant components, which represents a well-recognized limitation of water-soluble intumescent systems. The hydrophobic FHPL overlayer effectively mitigates this vulnerability by reducing water uptake at the coating surface, thereby extending the service lifetime of the flame-retardant functionality under humid or wet exposure conditions without compromising the underlying self-healing capacity, which as demonstrated above is activated selectively by direct water contact at damage sites rather than passive ambient humidity. To further verify the stability of hydrophobicity, we placed the treated samples at 25 °C and 90% relative humidity for 3 days. As shown in Figure S17a, the water contact angle (WCA) decreased from 122° to about 117°, still higher than the conventional hydrophobic threshold (WCA > 90°). This result confirms that the fluorocarbon coating derived from FHPL can still provide durable hydrophobicity under sustained high humidity conditions.

### 2.4. Fire Protective Performance of Coating

#### 2.4.1. Alcohol Burner Test with Infrared Thermal Imaging

**Figure 4:**
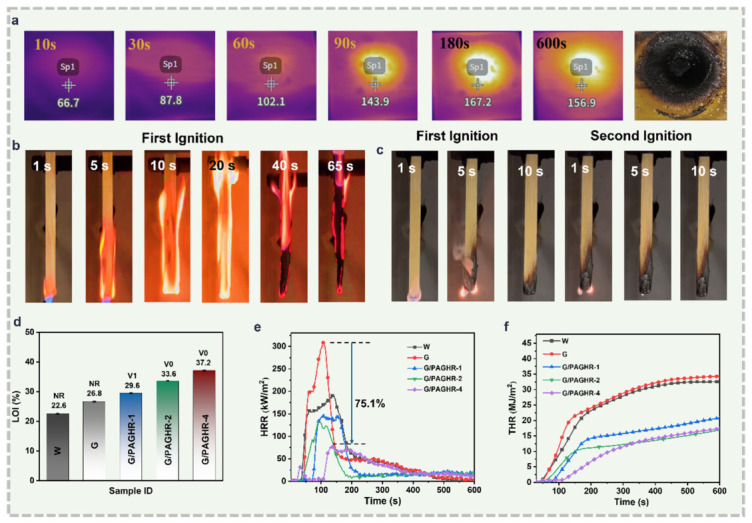
(**a**) Digital images for the burning behavior of coated wood under the alcohol burner flame for 600 s, along with their side temperature variation with time determined by the IR camera. Burning screenshots and residue for wood (**b**) and G/PAGHR-4 (**c**) during UL-94 testing. (**d**) LOI and UL94 burning test results of W, W/G, G/PAGHR-1, G/PAGHR-2, and G/PAGHR-4. HRR (**e**) and THR (**f**) curves of W, G, G/PAGHR-1, G/PAGHR-2, and G/PAGHR-4 in cone calorimetry tests.

To assess the fire resistance of the G/PAGHR-coated wood, a custom-built fire-testing apparatus was constructed (Figure S8a). Front-surface flame exposure was monitored by digital imaging, while back-surface temperature evolution was recorded simultaneously using an infrared thermal imager. The alcohol burner flame used in this setup reaches temperatures of up to 900 °C, providing a stringent thermal challenge representative of real fire scenarios. The flame response of the G/PAGHR-4-coated wood was first evaluated using an alcohol burner combined with infrared thermal imaging (Figure 4a). During 600 s of direct flame exposure, the side temperature of the coated sample increases from 67 °C at 10 s to 88 °C at 30 s, 102 °C at 60 s, and 167 °C at 180 s, and then slightly decreases to 157 °C at 600 s. The relatively slow increase in temperature indicates that the coating is able to retard heat transfer and protect the wood substrate under direct flame attack. By contrast, untreated wood shows a much faster temperature increase under the same conditions (Figure S8b), reaching 170 °C at 30 s, 239 °C at 40 s, 434 °C at 50 s, and 475 °C at 60 s, accompanied by severe burning and collapse of the exposed region. The big difference between untreated and coated wood indicates that the G/PAGHR-4 coating provides effective thermal shielding, which is consistent with its ability to form a stable protective residue during burning.

#### 2.4.2. UL-94 and LOI

The UL-94 burning behavior and LOI results are summarized in Figure 4b–d and Table S3. Untreated wood and G-coated wood do not achieve a rating in the UL-94 test, indicating poor self-extinguishing capability. The burning behavior of G during the UL-94 test is further shown in Figure S9. In contrast, the PAGHR-containing coatings show clear improvement with increasing flame-retardant loading. G/PAGHR-1 reaches a V-1 rating, while G/PAGHR-2 and G/PAGHR-4 achieve the V-0 rating. The LOI values increase from 22.6% for untreated wood to 26.8% for G, 29.6% for G/PAGHR-1, 33.6% for G/PAGHR-2, and 37.2% for G/PAGHR-4. The continuous increase in LOI with PAGHR content indicates that the hybrid phosphorus–nitrogen system plays an essential role in suppressing combustion. The G/PAGHR-2 and G/PAGHR-4 both passed the UL-94 V-0 classification. This is the highest rating available under the UL-94 V-0 classification. It confirms that the P–N synergistic mechanism in PAGHR works at the practical coating level. The ELF bond analysis predicted a staged decomposition sequence in PAGHR. The multi-stage DTG profiles confirmed this prediction experimentally. Together, these results show that molecular-level design translates directly into macroscopic flame suppression.

PAGHR substantially improves the fire safety of the gelatin-based coating. A higher PAGHR loading consistently produces stronger flame-retardant performance. This trend holds across all tested formulations from G/PAGHR-1 to G/PAGHR-4.

#### 2.4.3. Cone Calorimetry

Cone calorimetry was conducted under an external heat flux of 35 kW m^−2^ to further assess fire hazard. The corresponding data are listed in Table S5, and the pHRR and THR curves are shown in Figure 4e,f. Pure wood exhibited a pHRR of 197 kW m^−2^ and a THR of 33 MJ m^−2^. Coating with neat G alone worsened both values: pHRR increased to 309 kW m^−2^ and THR rose to 34 MJ m^−2^. This deterioration is attributed to the additional combustible organic mass introduced by the gelatin layer, which lacks intumescent protection. This result confirms that the coating matrix is not inert under fire conditions. In the absence of PAGHR, gelatin combustion measurably amplifies local heat release. Progressive incorporation of PAGHR systematically reduced both pHRR and THR. G/PAGHR-4 achieved the most pronounced pHRR reduction, recording a value of 77 kW m^−2^. This improvement is attributed to the early formation of an expansive, continuous phosphate-rich char. The char development is driven by two coordinated processes: NH_3_ release from piperazinium N–C cleavage at ~290 °C acts as a blowing agent, while PO_3_ species generated from C–O and P–C bond cleavage at higher temperatures serve as condensed-phase char catalysts. The THR values of G/PAGHR-2 and G/PAGHR-4 were nearly identical at 17 and 17 MJ m^−2^, respectively. This similarity reflects a saturation in char-forming capacity. Beyond 20 wt% PAGHR, the phosphorus content is sufficient to catalyse near-complete substrate char coverage. Additional PAGHR loading therefore primarily accelerates early char formation rather than further reducing the total combustible inventory. Where the initial fire growth rate is the primary concern, G/PAGHR-4 is the preferred formulation. Where total heat dose governs the hazard assessment, G/PAGHR-2 offers comparable THR suppression at lower material cost. Digital photographs of the post-combustion residues are shown in Figure S11c–f. The residues of PAGHR-containing samples are significantly more continuous and coherent than those of G alone. This observation further supports the conclusion that the improved fire performance is closely associated with enhanced char formation and residue integrity. In Figure S15a, W exhibits a moderate smoke production rate peak (pSPR) (≈0.008 m^2^/s) at 60–80 s, corresponding to active pyrolysis of wood organic constituents, followed by a rapid decline to near-zero after 200 s as combustible components are substantially consumed. G records the highest pSPR (≈0.033 m^2^/s) among all samples—approximately 4-fold higher than W—temporally coinciding with its pHRR (309.4 kW/m^2^) at approximately 100–120 s. This reflects concentrated incomplete combustion of pyrolysis volatiles under localized oxygen-deficient conditions created by intense flaming. A persistent post-peak SPR plateau of ≈0.009 m^2^/s sustained throughout 150–600 s indicates continued low-rate smoke generation from residual organic components. G/PAGHR series SPR characteristics evolve with increasing PAGHR loading as follows: G/PAGHR-1: pSPR reduced to ≈0.018 m^2^/s relative to G, but a multi-peak profile (peaks at ≈40 s, 80 s, and 100–160 s) indicates repeated local char layer rupture events releasing volatiles intermittently. The post-peak SPR remains elevated at ≈0.010–0.011 m^2^/s over 200–600 s, consistent with residual microcracks in SEM morphology and a relatively high I_D_/I_G_ ratio (2.63). G/PAGHR-2: Despite an initial peak of ≈0.024 m^2^/s at 30–50 s, rapid formation of an effective char barrier quickly suppresses subsequent smoke release; SPR decreases to ≈0.001–0.003 m^2^/s after 200 s, consistent with improved char continuity in SEM and I_D_/I_G_ = 2.49. G/PAGHR-4: SPR drops to near-zero after approximately 80 s and remains essentially zero throughout 100–600 s, reflecting the complete volatile barrier established by the dense, high-quality char layer (CY = 41.0 wt%; lowest ID/IG = 2.47). In Figure S15b, W reaches a TSR of ≈100 m^2^/m^2^ at 600 s; the curve levels off after 200 s, consistent with the near-zero post-200 s SPR. G accumulates the highest TSR (≈750 m^2^/m^2^), approximately 6.5-fold higher than W, driven by its sustained post-peak SPR plateau (≈0.009 m^2^/s) throughout the test duration. G/PAGHR-1 achieves a TSR of ≈730 m^2^/m^2^, nearly equivalent to G despite its substantially lower pSPR. The persistent post-peak SPR plateau (≈0.010–0.011 m^2^/s over 200–600 s) more than offsets the reduction in peak intensity, yielding a final TSR comparable to the unretarded G coating. This result demonstrates that reducing pSPR alone, without improving char layer integrity to suppress sustained post-peak smoke release, is insufficient to achieve meaningful total smoke rate (TSR) reduction. G/PAGHR-2 reaches a TSR of ≈200 m^2^/m^2^ at 600 s—a reduction of approximately 73.3% relative to G—reflecting the simultaneous improvement in both peak and post-peak SPR resulting from enhanced char layer quality at the PAGHR-2 loading level. G/PAGHR-4 achieves the lowest TSR of ≈20 m^2^/m^2^, representing reductions of approximately 80.0% relative to W (≈100 m^2^/m^2^) and 97.3% relative to G (≈750 m^2^/m^2^). Virtually the entire TSR contribution occurs within the brief initial combustion window (0–80 s); after approximately 80 s, near-complete smoke suppression is maintained for the remainder of the test.

### 2.5. Char Residue Characterization and Flame-Retardant Mechanism

#### 2.5.1. Raman Analysis of Char Structure

**Figure 5:**
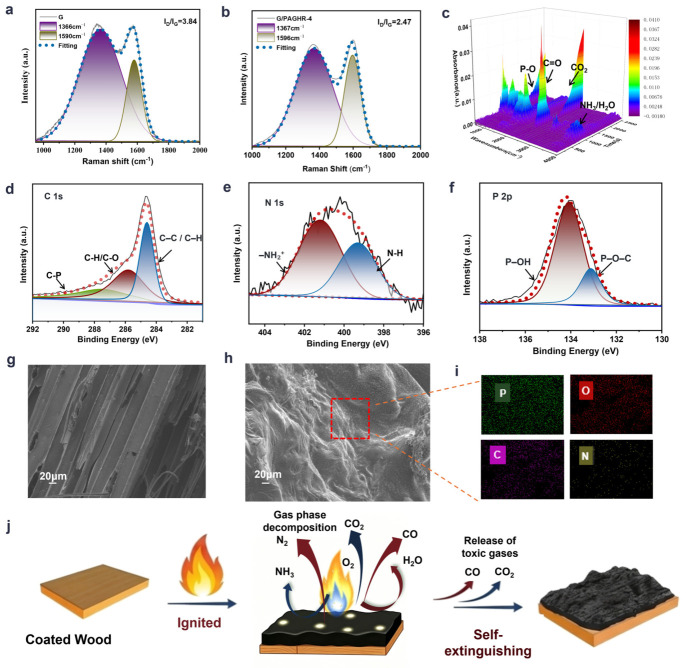
Raman spectra of the residual chars for the G (**a**) and G/PAGHR-4 (**b**). TG-IR spectra of thermal degradation products at different temperatures for the G/PAGHR (**c**). High-resolution C 1s (**d**), N 1s (**e**) and P 2p (**f**) XPS spectra of G/PAGHR-4. SEM photographs of the char residues for those coatings after cone testing: W (**g**), the surface (**h**) of G/PAGHR-4 with EDS elemental mapping (**i**). Schematic diagram of flame-retardant mechanism of Coated Wood (**j**).

Raman spectroscopy was used to evaluate the microstructural order of the residual char. As shown in Figure 5a,b and Figure S13, all samples exhibit the characteristic D and G bands of carbon materials. The calculated I_D_/I_G_ values are 3.84 for G, 2.63 for G/PAGHR-1, 2.49 for G/PAGHR-2, and 2.47 for G/PAGHR-4. The char residue of neat G exhibits the highest I_D_/I_G_ ratio of 3.84, indicating a highly disordered, defect-rich carbonaceous structure with minimal graphitic ordering. This is consistent with the absence of any phosphorus-catalyzed dehydration–aromatization pathway in the G-only system, resulting in a char of low structural integrity and correspondingly poor thermal barrier performance. The systematic decrease in I_D_/I_G_ with increasing PAGHR loading indicates a progressive enhancement of carbon structural ordering within the char, reflecting a higher degree of aromatic condensation and graphitic domain formation. The rigid inositol carbon skeleton of the PgP component, which persists to higher temperatures due to its strong C–C bond character, further serves as a structural template that guides the condensation of surrounding carbonaceous fragments into a more ordered graphitic network. This result agrees well with the increased residue yield observed in TGA and the improved residue integrity seen after cone calorimetry.

#### 2.5.2. TG-FTIR Analysis of Evolved Gaseous Products

In Figure S16a, at both 100 °C and 200 °C, the infrared spectra show essentially flat baselines across the entire wavenumber range, with no identifiable absorption peaks. This indicates that G/PAGHR-4 has not yet undergone appreciable thermal decomposition in this temperature range, and the material remains thermally stable. This observation is fully consistent with the TGA result of T_5%_ = 244.3 °C, confirming that no volatile pyrolysis products are released below 200 °C. At 300 °C, characteristic absorption features begin to emerge. A prominent absorption band appears in the 2300–2400 cm^−1^ region, assigned to the antisymmetric stretching vibration of CO_2_ (≈2349 cm^−1^), indicating that CO_2_ release commences at this temperature. This is consistent with the onset of decarboxylation and thermal cleavage of oxygen-containing functional groups such as C–O–C and C=O within the organic framework. Simultaneously, a group of absorption bands appears in the 1300–1600 cm^−1^ region, accompanied by a weak C–H stretching absorption near 3000 cm^−1^, attributable to small-molecule hydrocarbon volatiles produced during pyrolysis of the organic backbone. At 400 °C, all absorption intensities increase substantially relative to 300 °C. The CO_2_ band at approximately 2349 cm^−1^ reaches a notably higher intensity. An identifiable absorption peak near 950 cm^−1^, together with a deformation vibration band near 1630 cm^−1^, confirms significant NH_3_ release at this stage. The NH_3_ originates from preferential cleavage of C–N and C–H bonds within the piperazine-containing nitrogen component of PAGHR during thermal decomposition. This finding is in good agreement with the ELF analysis, which identified the piperazine N–C bond (ELF ≈ 0.7) as susceptible to rupture at moderate temperatures, thereby releasing NH_3_ prior to phosphate group decomposition. A broad absorption band in the 1000–1200 cm^−1^ region is also observed at 400 °C, assigned to P–O–C and P–O–P stretching vibrations. This indicates that phosphate ester groups begin to decompose at this stage, releasing phosphorus-containing gaseous intermediates into the gas phase. At 600 °C, the overall infrared absorbance drops to a low level. The C–H stretching region (approximately 2800–3000 cm^−1^) has essentially disappeared, while a broad absorption feature persists in the 1000–1300 cm^−1^ region, attributable to P–O vibrations associated with ongoing phosphate/polyphosphate condensation processes in the condensed phase. At 700 °C, only the CO_2_ absorption near 2349 cm^−1^ remains clearly identifiable; all other bands have largely disappeared. At 800 °C, the CO_2_ band reaches its highest intensity across all temperature stages, presenting a sharp and well-defined peak. This behavior indicates accelerated oxidation of residual carbon in the char layer at this temperature, with CO_2_ as the sole dominant gaseous product. The CO band near 2200 cm^−1^ at 700 and 800 °C: this absorption is assignable to CO stretching. At these high temperatures, residual carbon in the char undergoes partial oxidation under the trace oxygen present in the experimental atmosphere, generating CO as an incomplete combustion product.

During approximately 0–500 s (corresponding to approximately 100–200 °C), the entire map displays near-zero absorbance in the blue–purple color range, consistent with the thermally stable stage identified above. Between approximately 500 and 1000 s (approximately 200–350 °C), green-to-yellow signals begin to emerge near 2349 cm^−1^ and in the 1000–1600 cm^−1^ region, reflecting the gradual onset of volatile product release. The period from approximately 1000 to 1500 s (approximately 350–500 °C) represents the most intensive gas release stage of G/PAGHR-4, with strong absorbance signals appearing simultaneously across multiple wavenumber regions. This time window coincides with the main DTG decomposition peak temperature range, confirming that the majority of pyrolysis products are released within this stage. From approximately 1500 to 2000 s (approximately 500–650 °C), the overall absorbance decreases substantially, indicating a marked reduction in volatile release rate as the main decomposition event concludes. Between approximately 2000 and 2500 s (approximately 650–800 °C), a distinct red high-absorbance region reappears near 2349 cm^−1^, reaching a maximum absorbance of approximately 0.0410 a.u.—the highest value on the color scale. This isolated high-temperature CO_2_ signal is fully consistent with the 800 °C spectrum shown in Figure S16a, confirming high-temperature carbon oxidation as the source of this late-stage CO_2_ emission. Regarding the temporal sequence of gas release, NH_3_ characteristic absorption is most prominent during approximately 800–1500 s (approximately 250–450 °C), with peak intensity occurring between approximately 1000 and 1300 s (approximately 300–400 °C). This confirms that bulk NH_3_ release is concentrated in the low-to-moderate temperature decomposition stage, consistent with the “early gas release” mechanism proposed for the piperazine component of PAGHR. P–O stretching absorptions remain detectable over a broader window from approximately 1000 to 2000 s (approximately 300–650 °C), but at relatively lower intensity, indicating that phosphorus-containing volatile products are released over a more extended temperature range and in smaller quantities, with the majority of phosphate components retained in the condensed-phase char.

The TG-IR results provide direct experimental validation of the sequential multi-stage thermal response mechanism of G/PAGHR-4: NH_3_ is preferentially released at lower temperatures to provide gas-phase flame inhibition, followed by phosphate-related decomposition at higher temperatures to promote condensed-phase char formation. This release sequence is in close agreement with the bond dissociation order predicted by ELF calculations and supports the proposed phosphorus–nitrogen synergistic flame retardancy mechanism of the G/PAGHR system.

#### 2.5.3. XPS Analysis of Char Residue

XPS survey spectra reveal distinct elemental compositions between the two char residues (Figure S14a,c). The G char exhibits only C 1s (Figure S14d), N 1s (Figure S14e), and a weak O 1s (Figure S14f) signal, consistent with the peptide backbone of gelatin. In contrast, G/PAGHR-4 char shows pronounced N 1s and P 2p signals, confirming the retention of phosphorus- and nitrogen-containing species in the condensed-phase char and defining the characteristic of effective condensed-phase flame retardancy. O 1s (Figure S14b). Three oxygen species are identified: C–O–C/P–O–C/P–O–P (~533.0 eV, residual PEG ether linkages/cross-linked phosphate networks), P–O–C/P–O–P (~532.0 eV, cross-linked phosphate networks), P–OH/C–OH (~533.5 eV, partially hydrolyzed phosphate), and P=O (~531.0 eV, meta-phosphate/polyphosphate). The coexistence of these species confirms the formation of a phosphate-rich glassy barrier layer during combustion. C 1s (Figure 5d). Peaks at ~284.8 eV (C–C/C–H), ~285.5 eV (C–P), and ~286.2 eV (C–H/C–C) are resolved. The presence of C–P bonding indicates covalent integration of phosphorus into the carbonaceous char matrix, enhancing its thermal stability. N 1s (Figure 5e). Two components at ~399.5 eV (–NH_2_/N–H) and ~401.5 eV (N–H) are detected, with no significant NO_X_ signal above 402 eV. This suggests preferential release of nitrogen-containing fragments into the gas phase, contributing to radical quenching and fuel dilution. P 2p (Figure 5f). Peaks at ~133.5 eV (P–OH) and ~134.5 eV (P–O–C) confirm the formation of phosphate ester linkages and acidic phosphate intermediates within the char, consistent with a poly-phosphoric acid-driven dehydration mechanism.

The XPS data collectively demonstrate that PAGHR fundamentally redirects the thermal degradation pathway of the hybrid coating. While G alone undergoes uncontrolled oxidative decomposition, yielding a structurally deficient char, the incorporation of PAGHR promotes polyphosphoric acid-catalyzed dehydration, reinforces the char skeleton through P–O–P and P–O–C cross-linking, and simultaneously supplements gas-phase flame inhibition via nitrogen-containing volatile release. This dual condensed-phase/gas-phase mechanism underpins the superior flame-retardant performance of the G/PAGHR-4 system.

#### 2.5.4. Morphology of Residual Char

Char morphology after cone calorimetry testing was examined by SEM to evaluate the structural integrity of the residues (Figure 5g–i). Untreated wood yielded a loose, discontinuous char with evident structural collapse (Figure 5g). In contrast, G/PAGHR-4 produced a relatively continuous and intact surface morphology in the observed regions. (Figure 5h). EDS elemental mapping of the G/PAGHR-4 char confirms the uniform distribution of P, O, C, and N across the residue surface (Figure 5i), consistent with the formation of a phosphorus-enriched intumescent barrier. The char residues of G/PAGHR-1 and G/PAGHR-2 shown in Figure S12 also exhibit expanded and continuous structures, although their compactness is lower than that of G/PAGHR-4. These observations indicate that PAGHR facilitates the formation of an effective protective residue during burning.

#### 2.5.5. Flame-Retardant Mechanism

The convergent evidence from ^31^P NMR, FTIR, XPS, Raman spectroscopy, SEM, EDS, TGA/DTG, and ELF analysis supports a self-consistent two-phase, three-stage flame-retardant mechanism, as summarised in Figure 5f. Stage 1: Gas-phase inhibition (below ~290 °C). In the early heating stage, thermally labile N–C bonds (ELF ≈ 0.7) within the piperazinium units of HEPHR cleave preferentially. This releases NH_3_ and nitrogen-containing volatile fragments into the combustion zone, diluting flammable gases and interrupting radical chain propagation. The early DTG decomposition shoulder at ~290 °C and the retention of –NH_2_^+^ and N–H species in the char XPS N 1s spectrum both corroborate this assignment. Stage 2: Char initiation and intumescence (~290–400 °C). As temperature increases, polar C–O bonds at the phosphate–inositol interface of PgP (ELF → 1.0) cleave, releasing PO_3_ species that catalyse dehydration and cross-linking of the organic matrix to initiate char formation. Concurrently, NH_3_ generated in Stage 1 acts as a blowing agent, expanding the softening char into an intumescent foam that maximises thermal insulation thickness. Stage 3: Char consolidation (above ~400 °C). At elevated temperatures, the stronger P–C bonds of the HEDP bisphosphonate units (ELF ≈ 0.8) contribute additional PO_3_ species, driving the formation of thermally stable polyphosphate glass. This cements an inorganic–organic composite char matrix, directly evidenced by the dominant P–O–C/P–O–P component in the O 1s XPS signal and the P–O–C component in the P 2p XPS spectrum. The resulting char exhibits a layered architecture: an outer inorganic polyphosphate glassy layer overlying an inner graphitised carbonaceous foam (I_D_/I_G_ = 2.47). This structure physically impedes bidirectional heat and mass transfer, blocking both radiative and conductive heat flux while suppressing outward diffusion of combustible pyrolysis gases. The loading-dependent flame-retardant metrics collectively validate this multi-mechanism design [54]. LOI increased from 22.6% for uncoated wood (W) to 37.2% for G/PAGHR-4. The UL-94 rating advanced from NR to V-0. Relative to the control G, pHRR and THR were reduced by 75.1% and 49.6%, respectively. Progressive char graphitization improvement was also confirmed, with I_D_/I_G_ decreasing from 3.84 to 2.47. These trends are directly traceable to the hierarchical bond-strength ordering within the PAGHR molecular architecture.

## 3. Conclusions

A bio-derived multifunctional coating for wood was successfully developed through the hierarchical ionic assembly of a phosphorylated phytic acid–poly(ethylene glycol) conjugate (PgP) and a piperazinium hydroxyethylidene diphosphonate salt (HEFER), followed by cross-linking with gelatin and surface fluorination.

Synthesis and Structure: DFT calculations confirmed the (1,3,5) substitution pattern of PgP as the lowest-energy configuration; FTIR, NMR, and ELF analyses collectively verified the successful construction of the P–N ionic hybrid network in PAGHR.

Thermal Stability: Incorporation of PAGHR increased the residual char yield at 700 °C from 17.8 wt% (neat G) to 41.0 wt% (G/PAGHR-4).

Flame Retardancy: G/PAGHR-4 achieved an LOI of 37.2% and passed the UL-94 V-0 rating; pHRR and THR were reduced by 75.1% and 50.0%, respectively, compared to the neat G control.

Self-Healing Performance: Macroscopic scratch healing was achieved within 40 min at ambient temperature through moisture-mediated dynamic hydrogen bond reconstruction.

Optical Transparency: All G/PAGHR formulations maintained high transmittance in the 400–800 nm visible light range (specific values to be supplemented in the revised manuscript).

Surface Hydrophobicity: Following FHPL post-treatment, the water contact angle increased from 59° (untreated wood) to 122°, effectively suppressing moisture-induced leaching of the phosphorus–nitrogen components.

Design Strategy: This work demonstrates that the hierarchical ionic assembly of phytic acid-based phosphate ester, bisphosphonate–piperazine salt, and gelatin into a unified bio-based coating can simultaneously achieve flame retardancy, transparency, self-healing, and hydrophobicity, providing a generalizable molecular design strategy for multifunctional bio-based protective coatings.

## Data Availability

The original contributions presented in this study are included in the article/Supplementary Material. Further inquiries can be directed to the corresponding authors.

## Supplementary Materials

The following supporting information can be downloaded at: https://www.mdpi.com/article/10.3390/polym18121497/s1. Figure S1: The synthetic scheme of PgP; Figure S2: The synthetic scheme of HEPHR; Figure S3: The synthetic scheme of PAGHR; Figure S4: The energy calculations were performed for three coordination modes of polyethylene glycol (PEG) grafted onto phytic acid: a (1,2,3), b (1,3,4), and c (1,3,5); Figure S5: Electron localization function of (a) PgP and (b) HEPHR, (c) Model before HEPHR structure optimization (d) Model after optimization; Figure S6: 1H (a) NMR spectra of PAGHR and HEPHR. 31P NMR (b) spectra of PAGHR and HEPHR; Figure S7: The corresponding EDS mapping images of N; Figure S8: (a) The homemade setup for determining the TST of steel plates. Digital photos of W (b) after vertical combustion test; Figure S9: Burning screenshots and residue for G (a) during UL-94 testing; Figure S10: The digital photos of the transparency of the G (a), G/PAGHR-1 (b) and G/PAGHR-2 (c) coatings.(The G/PAGHR film was placed directly in front of the flower.); Figure S11: (a,b) Surface morphologies of wood samples treated with different formulations. (c–f) Residual char morphologies of the corresponding treated wood samples after combustion; Figure S12: SEM photographs of the char residues for those coatings after cone calorimetry tests: G/PAGHR-1 (a) and G/PAGHR-2 (b); Figure S13: Raman spectra of the residual chars for the G/PAGHR-1 (a) and G/PAGHR-2 (b); Figure S14: XPS survey spectra of the char residues for the G/PAGHR-4 (a). High-resolution O 1s (b) XPS spectra of G/PAGHR-4. XPS survey spectra of the char residues for the G (c). High-resolution C 1s (d), N 1s (e) and O 1s (f) XPS spectra of G; Figure S15: Smoke production rate (SPR, m^2^/s) and total smoke release (TSR, m^2^/m^2^) as a function of time (0–600 s) for W, G, and G/PAGHR series coated wood samples during cone calorimeter testing (heat flux: 35 kW/m^2^). (a) SPR curves; (b) TSR curves; Figure S16. TG-IR combined test results of G/PAGHR-4. (a) Overlay of infrared absorption spectra of pyrolysis gases at different temperatures (100~800 °C); Figure S17. The water contact angle of the treated wood after aging test (a); Figure S18. Thermogravimetric analysis of PAGHR under nitrogen atmosphere. (a) TGA curve and (b) DTG curve; Table S1: Formula of the gelatin-based intumescent flame-retardant coatings; Table S2: The total energies obtained from DFT geometry optimization are summarized; Table S3: LOI and UL-94 vertical burning test results for the uncoated and coated woods; Table S4: Thermal properties of the G and G/PAGHR coatings under air and nitrogen respectively; Table S5: Cone calorimetry data for the uncoated and coated woods.
