# Supplementary material for: A Self-Healing, Transparent, and Hydrophobic Flame-Retardant Coating for Wood Based on Bio-Derived Flame Retardants and Fluorosilane Surface Treatment
Source: Polymers (Basel). 2026 Jun 15;18(12):1497. doi: 10.3390/polym18121497 (PMC13307277; doi:10.3390/polym18121497)
Supplement: Supplementary file 1 [file polymers-18-01497-s001.zip › polymers-4351215-supplementary.pdf]

Supporting Information

Figure S1. The synthetic scheme of PgP.

Figure S2. The synthetic scheme of HEPHR.

Figure S3. The synthetic scheme of PAGHR.

Figure S4. The energy calculations were performed for three coordination modes of polyethylene glycol (PEG) grafted onto phytic acid: a (1,2,3), b (1,3,4), and c (1,3,5).

Figure S5. Electron localization function of (a) PgP and (b) HEPHR.

Figure S6. 1H (a) NMR spectra of PAGHR and HEPHR. 31P NMR (b) spectra of PAGHR and HEPHR.

Figure S7. The corresponding EDS mapping images of N.

Figure S8. (a) The homemade setup for determining the TST of steel plates. Digital photos of W (b) after vertical combustion test.

Figure S9. Burning screenshots and residue for G (a) during UL-94 testing.

Figure S10. The digital photos of the transparency of the G (a), G/PAGHR-1 (b) and G/PAGHR-2 (c) coatings. (The G/PAGHR film was placed directly in front of the flower.)

Figure S11. (a–b) Surface morphologies of wood samples treated with different formulations. (c–f) Residual char morphologies of the corresponding treated wood samples: G (c), G/PAGHR-1 (d), G/PAGHR-2 (e) and G/PAGHR-4 (f) after combustion.

Figure S12. SEM photographs of the char residues for those coatings after cone calorimetry tests: G/PAGHR-1 (a) and G/PAGHR-2 (b).

Figure S13. Raman spectra of the residual chars for the G/PAGHR-1 (a) and G/PAGHR-2(b).

Figure S14. XPS survey spectra of the char residues for the G (c) and G/PAGHR-4 (a). High-resolution O 1s (b) XPS spectra of G/PAGHR-4. High-resolution C 1s (d), N 1s (e) and O 1s (f) XPS spectra of G.

Figure S15. Smoke production rate (SPR, m^2/s) and total smoke release (TSR, m^2/m^2) as a function of time (0–600 s) for W, G, and G/PAGHR series coated wood samples during cone calorimeter testing (heat flux: 35 kW/m^2). (a) SPR curves; (b) TSR curves.

Figure S16.TG-IR combined test results of G/PAGHR-4. (a) Overlay of infrared absorption spectra of pyrolysis gases at different temperatures (100~800°C).

Figure S17. The water contact angle of the treated wood after the aging test (a).

Figure S18. Thermogravimetric analysis of PAGHR under nitrogen atmosphere. (a) TGA curve and (b) DTG curve.

Table S1. Formula of the gelatin-based intumescent flame-retardant coatings.

Table S2. The total energies obtained from DFT geometry optimization are summarized.

Table S3. LOI and UL-94 vertical burning test results for the uncoated and coated woods.

Table S4. Thermal properties of the G and G/PAGHR coatings under air and nitrogen respectively.

Table S5. Cone calorimetry data for the uncoated and coated woods.

1. Materials and Methods

1.1 Materials

Phytic acid (PA, 70% aqueous solution), etidronic acid (HEDP), piperazine (P, ⩾ 99%(T)), polyethylene glycol (Mn: 200 g mol^{-1}, PEG-200), gelatin (G), 1H,1H,2H,2H-Perfluorodecyltrichlorosilane (FHPL) and isopropanol (IPA) were commercially obtained from Shanghai Aladdin Biochemical Technology Co., Ltd. (Shanghai, China) and used as received without further purification. The wood substrate was supplied by Luban Wooden Manual Model Materials Factory.

1.2 Synthesis of Phytate-Based Flame Retardant (PgP)

PA and PEG-200 were introduced into a three-necked round-bottom flask at a molar ratio of 1:3. The reaction mixture was mechanically stirred at 130 °C for 5 h under nitrogen atmosphere to minimize oxidative side reactions. After the reaction, a dark brown viscous product was obtained and denoted as PgP (**Figure S1**). The reaction mixture constitutes the desired esterification product and no insoluble byproducts were observed. The isolated yield based on the combined mass of PA and PEG-200 was approximately 93 wt%, with the mass loss attributable primarily to water generated during esterification condensation. No additional purification was required.

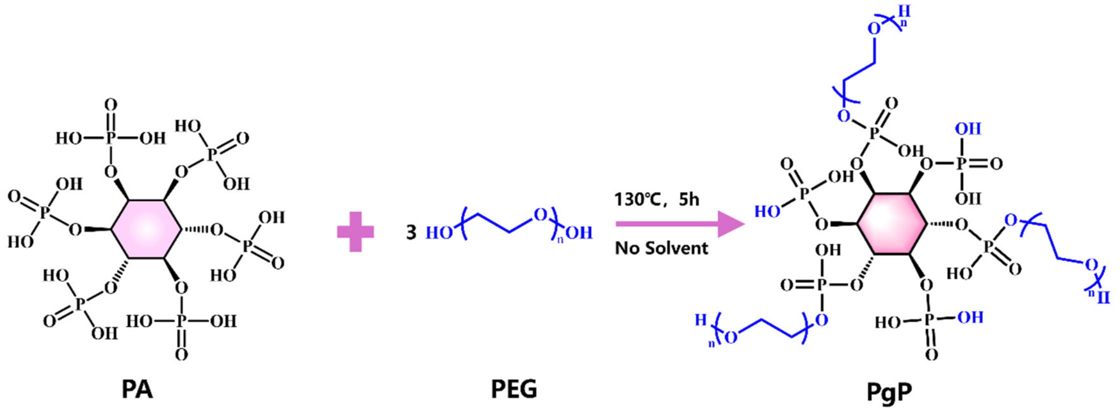

Figure S1. The synthetic scheme of PgP.

1.3 Synthesis of Piperazine–HEDP Salt (HEPHR)

Piperazine (0.1 mol) was dissolved in 200 g of anhydrous ethanol under continuous magnetic stirring at ambient temperature. Subsequently, an equimolar quantity of etidronic acid (HEDP, 0.1 mol) was introduced into the solution at a piperazine-to-HEDP molar ratio of 1:1, and the resulting mixture was stirred at 50 °C for 6 h. The precipitated white solid was collected by vacuum filtration, washed thoroughly with anhydrous ethanol to remove residual reactants, and dried to constant weight under reduced pressure. The obtained white crystalline product was designated as HEPHR **(Figure S2)**. The isolated yield was 85%. The main potential byproduct is excess unreacted piperazine, which is removed by the ethanol washing steps; its presence in trace amounts would not affect the ionic assembly with HEDP.

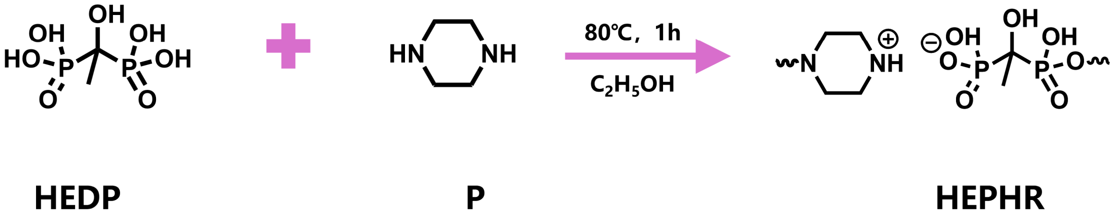

Figure S2. The synthetic scheme of HEPHR.

1.4 Preparation of Gelatin-Based Intumescent Flame-Retardant Coating (PAGHR)

PgP and HEPHR were introduced into a three-neck flask at a molar ratio of 1:3 under nitrogen atmosphere. The mixture was reacted at 80 °C for 1 h to obtain a homogeneous solution. The resulting solution was then placed in an oven at 90 °C for 24 h to afford a uniform brown product, which was denoted as PAGHR **(Figure S3)**. The overall yield of PAGHR based on the combined input masses was approximately 96 wt%, consistent with the ionic assembly mechanism that does not generate small-molecule byproducts.

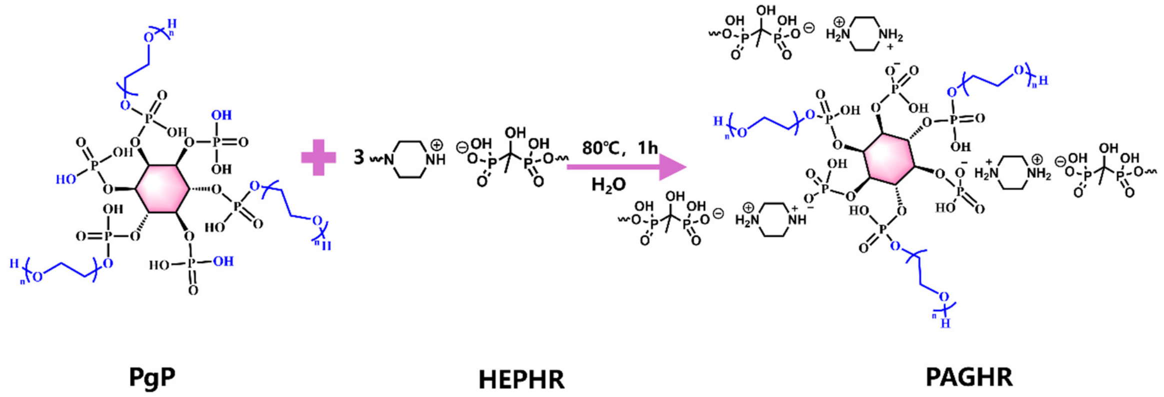

Figure S3. The synthetic scheme of PAGHR.

1.5 Preparation of G/PAGHR hybrid coatings

G and PAGHR were combined according to the formulations listed in Table S1 to prepare the coating compositions. Gelatin was first dissolved in water under stirring for 30 min until a clear solution was obtained, and PAGHR was then added after complete dissolution of gelatin. The resulting mixtures were used to prepare the coating formulations, which were denoted as G, G/PAGHR-1, G/PAGHR-2, and G/PAGHR-4 according to PAGHR content.

Table S1. Formula of the gelatin-based intumescent flame-retardant coatings

**Sample**	G (wt%)	PAGHR(wt%)	G/PAGHR（%）
G	100	0	—
G/PAGHR-1	90	10	10
G/PAGHR-2	80	20	20
G/PAGHR-4	60	40	40

1.6 Preparation of wood coated wood samples

The G/PAGHR composite coating was deposited onto wood substrates via a blade-coating technique at a controlled coating areal density of 0.02 g cm^{-2}. The coated specimens were subsequently dried in a forced-air oven at 60 °C for 1 h to facilitate solvent evaporation and film formation. Residual bound water in gelatin networks is difficult to remove without thermal degradation of the protein matrix. To impart hydrophobic character to the coating surface, a solution of 1H,1H,2H,2H-perfluorodecyltrichlorosilane (0.1 g) dissolved in isopropanol (9.9 g) was uniformly applied onto the G/PAGHR coating surface. The treated specimens were then thermally cured in an oven at 85 °C for 1 h. This fluorosilane treatment and curing cycle was repeated to obtain a stable hydrophobic surface.

1.7 Characterizations and measurements

^{1}H and ^{31}P nuclear magnetic resonance (NMR) analysis were conducted by a Bruker AVANCE III NMR spectrometer using deuterated chloroform as solvent. ^{1}H NMR: Tetramethylsilane (TMS) was used as the internal reference. Chemical shifts are reported in ppm. ^{31}P NMR: 85% aqueous H3PO4 was used as an external reference. Chemical shifts are reported in ppm.

Fourier-transform infrared (FTIR) spectra were measured by a Thermo Fisher Nicolet 6700

FTIR spectrophotometer. The scanning time was 32 and the wavenumber ranged from 500 to 4000 cm^{-1}.

Density functional theory (DFT) calculations were performed using the CASTEP module within Materials Studio. The generalized gradient approximation (GGA) with the Perdew–Burke–Ernzerhof (PBE) functional was employed. A plane-wave cutoff energy of 500 eV was used based on convergence tests. The convergence criteria for the self-consistent field (SCF) calculations were set to 1.0×10^{-6} eV/atom for energy and 1.0×10^{-5} e/$Å^3$ for electron density. Geometric optimizations utilized the BFGS algorithm with convergence thresholds of 0.03 eV/Å for atomic forces and 0.05 GPa for stress. A Monkhorst–Pack k-point mesh of 2×2×1 was used for Brillouin zone sampling. A vacuum layer of 15 Å was applied perpendicular to the surface to eliminate periodic interactions.

The thermal degradation properties of the PAGHR films were studied using a TAQ5000 thermal analyzer instrument (USA). The samples were heated from room temperature to 800 °C at a heating rate of 20 °C/min under nitrogen atmosphere, and flow rate of 50 mL/min. The thermogravimetric analysis/infrared spectrometry (TG-FTIR) technique was applied to detect the evolution of gaseous products during heating. The samples were performed on a Q5000 IR thermogravimetric analyzer interfaced to a Nicolet 6700 FTIR spectrophotometer. About 5.0 mg of the sample was heated from room temperature to 800 °C at a heating rate of 20 °C/min under nitrogen atmosphere. TG-FTIR: FTIR cell resolution 4 cm-1, 16 scans per spectrum, spectral range 400-4000 cm^{-1}, transfer line maintained at 220 °C to prevent condensation of volatile products.

The spectral reflectance ρ (λ) and transmittance c in the wavelength range of ultraviolet,

visible light, and near-infrared (0.3~2.5 μm) were determined by polytetrafluoroethylene integrating sphere UV-Visible-near-infrared spectrophotometer (Hitachi U-4100, Japan).

The LOI test was carried out on an HC-2 instrument (Jiangning, China) following ASTM D2863-97. UL-94 vertical burning test was conducted on a CFZ-2-type instrument (Jiangning, China) according to the GB/T 8333-2008.

A cone calorimeter (Suzhou Yangyi Vouch Testing Technology Co., Ltd.) was used to analyze the combustion behavior of the uncoated and coated wood based on the principle of oxygen consumption according to the ASTME1354/ISO5660. The radiation heat flux used for testing is 35 kW/m^2. Cone calorimetry measurements were based on the oxygen consumption principle, using the Huggett constant (E = 13.1 MJ/kg O_2) to convert oxygen depletion into heat release rate. The sample size used for testing is 100×100×3 mm^3. The char residue of each sample after cone calorimetry tests was used for scanning electron microscope (SEM), Raman spectroscopy and X-ray photoelectron spectroscopy (XPS) tests.

Raman analysis conditions: excitation wavelength 532 nm, laser power ~5 mW, 50x objective, acquisition time 10 s, 3 accumulations, spectral range 800-2000 cm^{-1}.

The microstructure and elemental mappings of char residue were obtained by using an EVO MA15 scanning electron microscope (SEM). The energy dispersive X-ray spectroscopy (EDS) was conducted to analyze the elemental contents of the char residues, which was carried out on the same SEM. The microstructure of the char residues was observed by Laser Confocal Roman Microscope (Lab RAM HR Evolution, France) with a 514.5 nm argon ion laser. Thermo ESCALAB 250Xi X-ray photoelectron spectroscopy

(XPS) was used to obtain the element composition of the char residue, which using monochromatic Al Ka (hv =1486.6 eV) as the X-ray excitation source.

An infrared camera (FLIR T420) was utilized to record surface temperatures of the control and treated wood during the flame-burning test. The sample size used for testing is 100×100×3 mm^3.

A UV-Vis-NIR spectrophotometer with a PTFE integrating sphere was used. The measurement wavelength range was 300-2500 nm with a spectral resolution of 2 nm. A blank PTFE plate served as the reference.

The water repellency of the coated wood was evaluated by the water contact angle (CA) of the surface, which was measured by a contact angle meter (JC2000D, Shanghai Zhongchen Powereach Company, China) at room temperature. The water CA value was recorded 15 s after a water droplet (5 μL) was placed on the surface of the sample, and the average CA value was obtained by measuring more than five positions for each sample. The treated G/PAGHR-4 by FHPL. FHPL is used exclusively as a post-treatment step to impart surface hydrophobicity. It is applied only to samples specifically intended for hydrophobicity assessment, and is entirely absent from all other characterization tests, including flame retardancy, self-healing, optical transmittance, and thermal analysis.

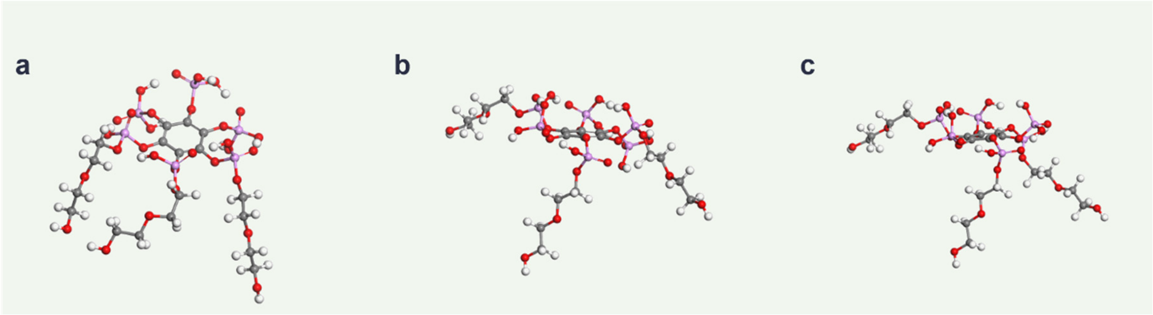

Figure S4. The energy calculations were performed for three coordination modes of polyethylene glycol (PEG) grafted onto phytic acid: a (1,2,3), b (1,3,4), and c (1,3,5).

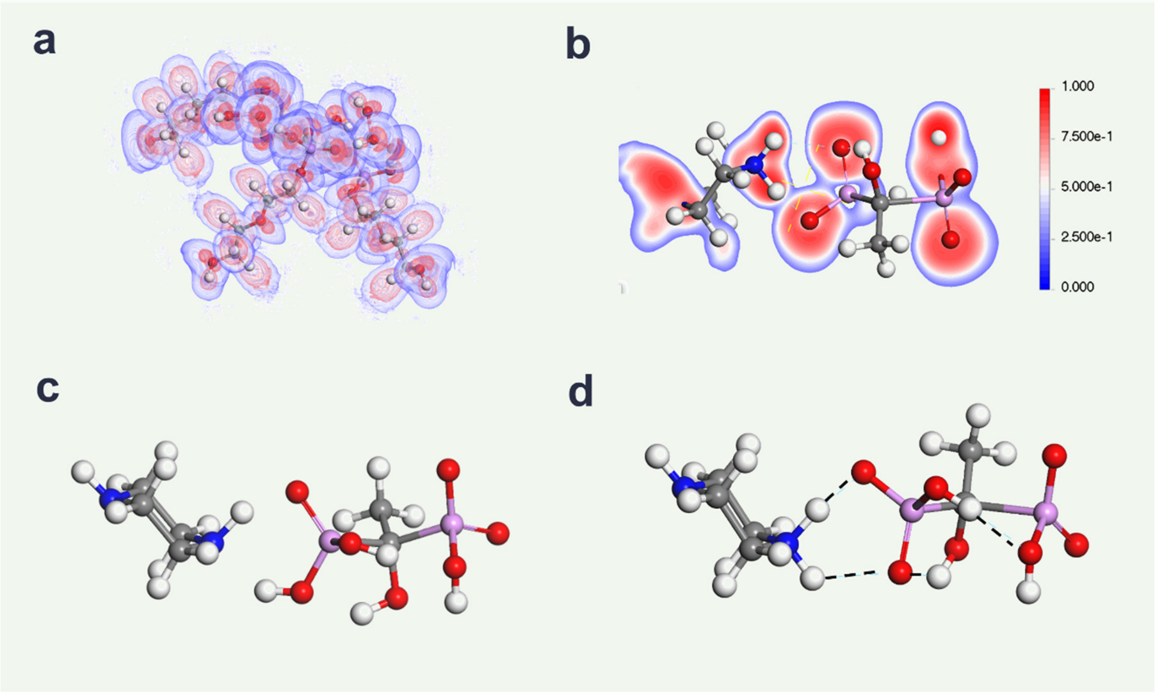

Figure S5. Electron localization function of (a) PgP and (b) HEPHR. (c) Model before HEPHR structure optimization. (d) Model after optimization.

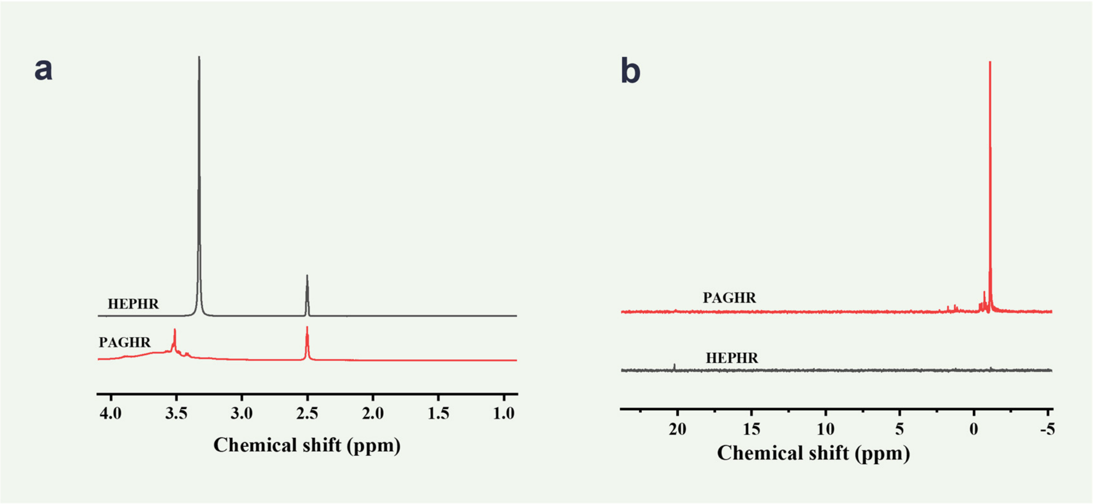

Figure S6. ^{1}H (a) NMR spectra of PAGHR and HEPHR. ^{31}P NMR (b) spectra of PAGHR and HEPHR.

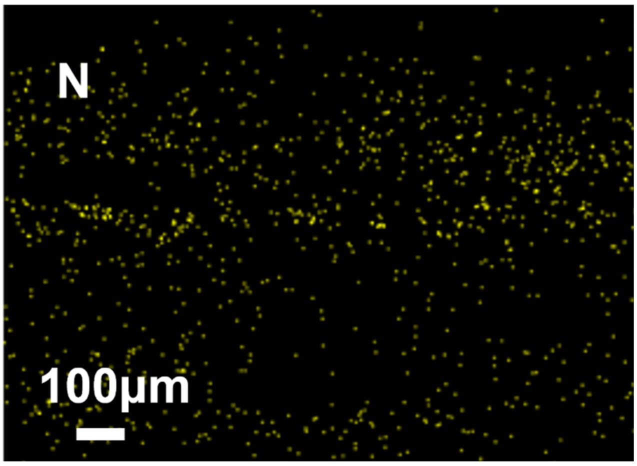

Figure S7. The corresponding EDS mapping images of N.

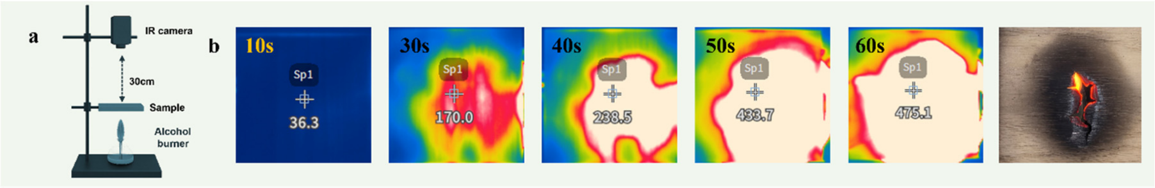

Figure S8. (a) The homemade setup for determining the TST of steel plates. Digital photos of W (b) after vertical combustion test.

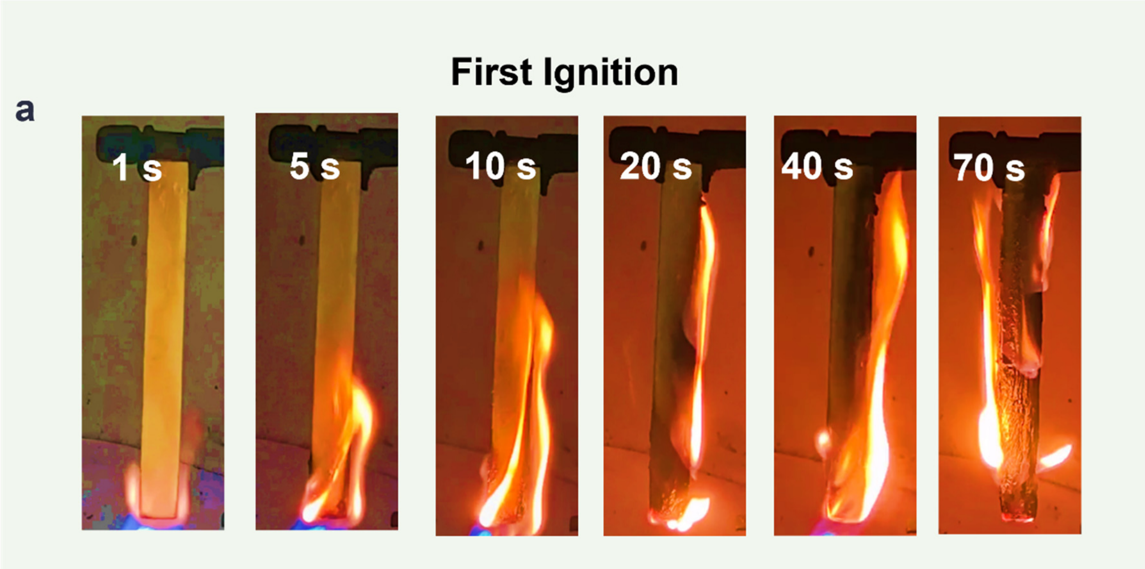

Figure S9. Burning screenshots and residue for G (a) during UL-94 testing.

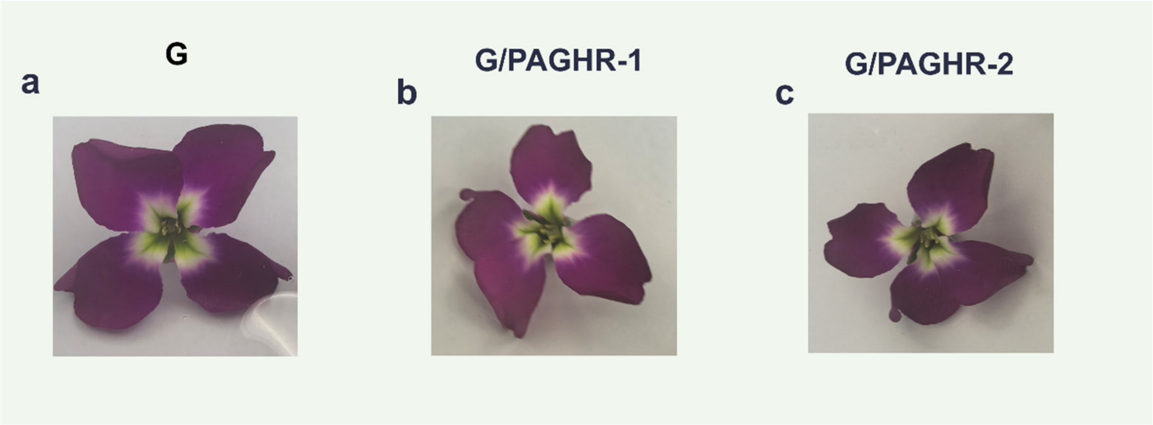

Figure S10. The digital photos of the transparency of the G (a), G/PAGHR-1 (b) and G/PAGHR-2 (c) coatings. (The G/PAGHR film was placed directly in front of the flower.)

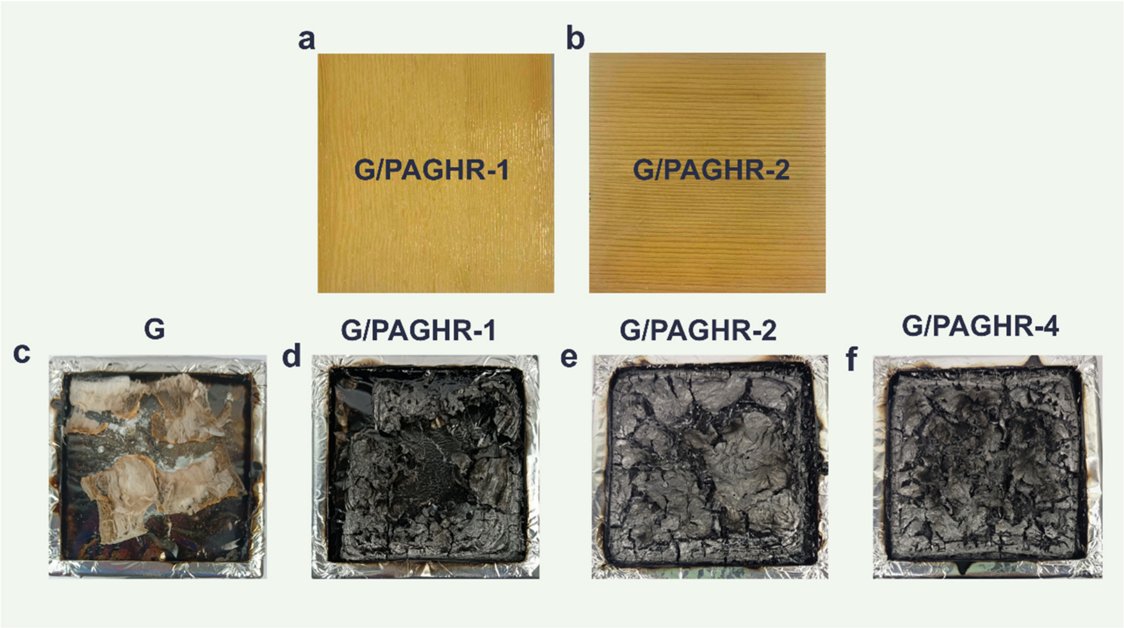

Figure S11. (a–b) Surface morphologies of wood samples treated with different formulations. (c–f) Residual char morphologies of the corresponding treated wood samples: G (c), G/PAGHR-1 (d), G/PAGHR-2 (e) and G/PAGHR-4 (f) after combustion.

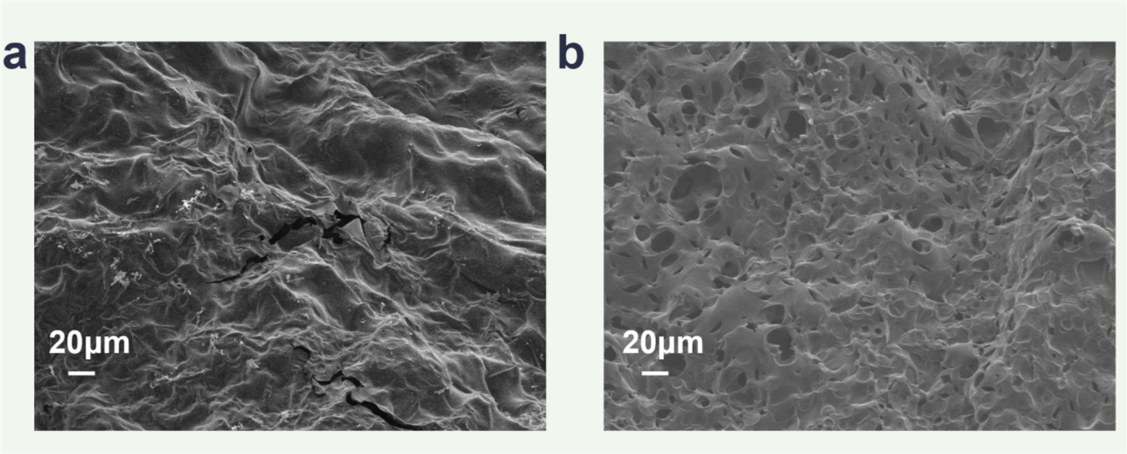

Figure S12. SEM photographs of the char residues for those coatings after cone calorimetry tests: G/PAGHR-1 (a) and G/PAGHR-2 (b).

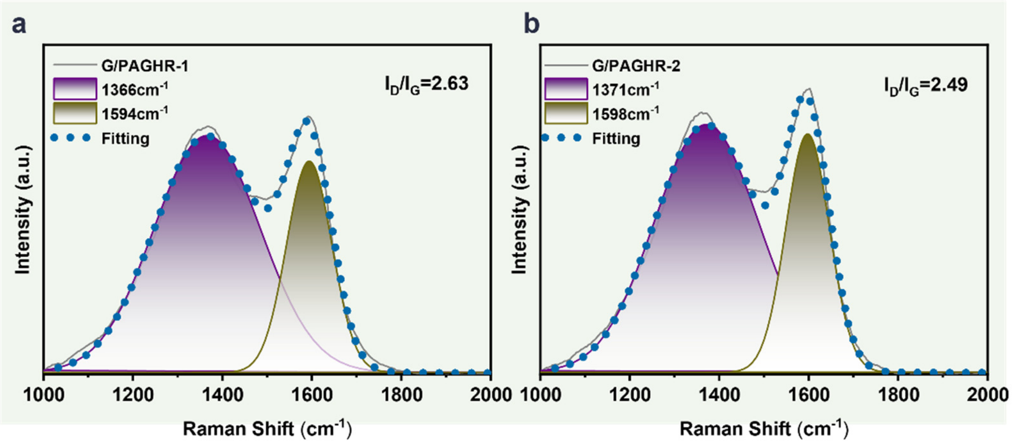

Figure S13. Raman spectra of the residual chars for the G/PAGHR-1 (a) and G/PAGHR-2(b).

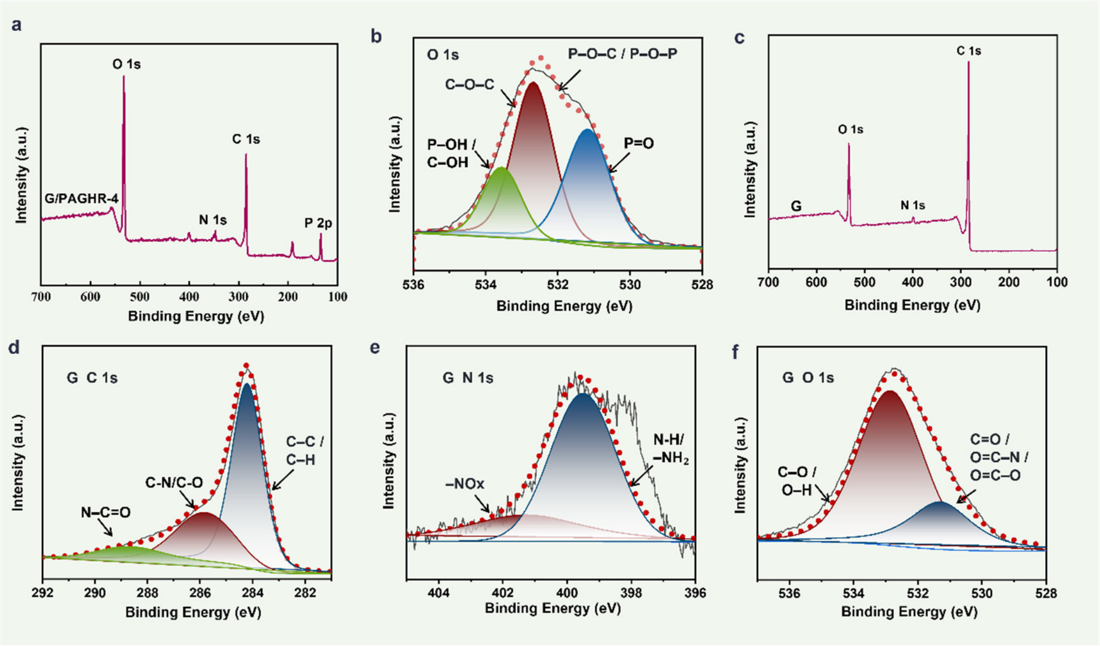

Figure S14. XPS survey spectra of the char residues for the G/PAGHR-4 (a). High-resolution O 1s (b) XPS spectra of G/PAGHR-4. XPS survey spectra of the char residues for the G (c). High-resolution C 1s (d), N 1s (e) and O 1s (f) XPS spectra of G.

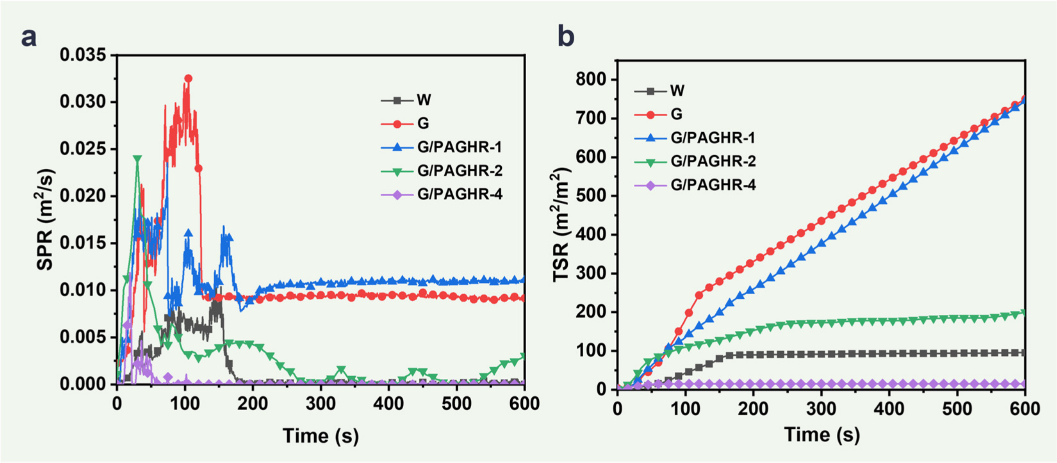

Figure S15. Smoke production rate (SPR, m^2/s) and total smoke release (TSR, m^2/m^2) as a function of time (0–600 s) for W, G, and G/PAGHR series coated wood samples during cone calorimeter testing (heat flux: 35 kW/m^2). (a) SPR curves; (b) TSR curves.

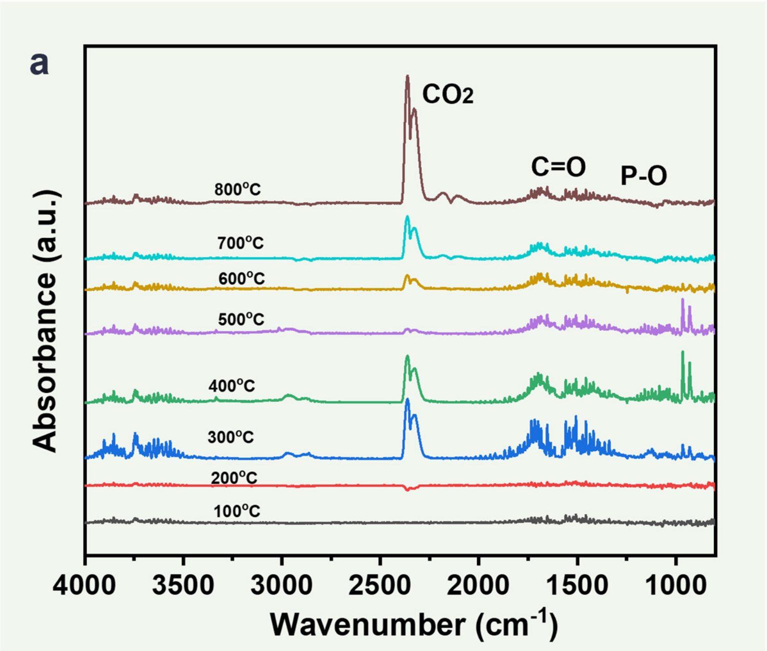

Figure S16.TG-IR combined test results of G/PAGHR-4. (a) Overlay of infrared absorption spectra of pyrolysis gases at different temperatures (100~800°C).

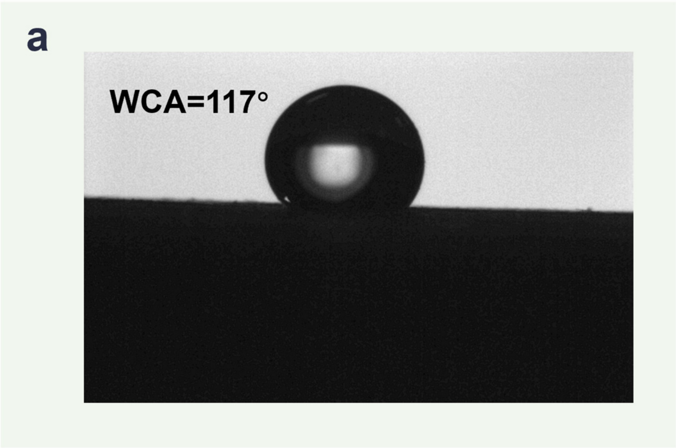

Figure S17. The water contact angle of the treated wood after the aging test (a).

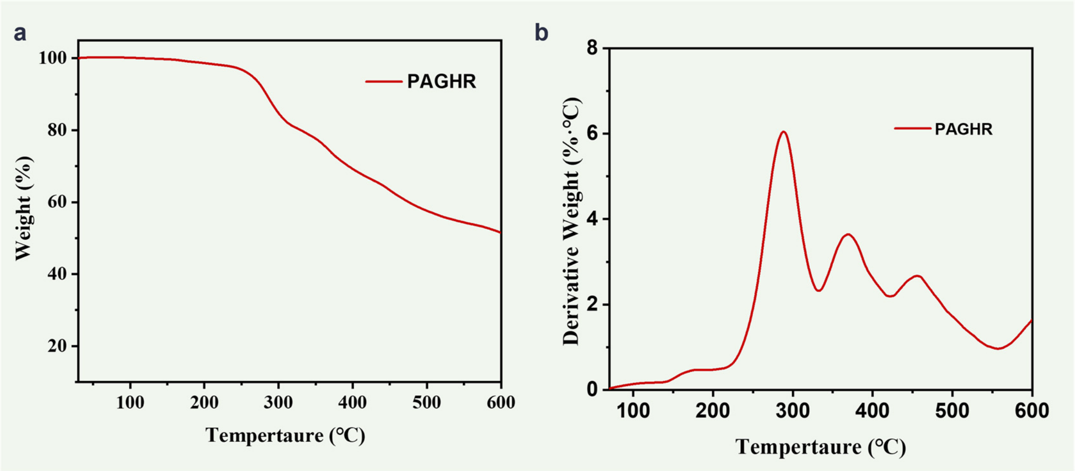

Figure S18. Thermogravimetric analysis of PAGHR under nitrogen atmosphere. (a) TGA curve and (b) DTG curve.

Table S2. The total energies obtained from DFT geometry optimization are summarized.

Configuration	Substitution Pattern	Total Energy (eV)
a	(1,2,3)	−17932.40635
b	(1,3,4)	−17932.06046
c	(1,3,5)	−17932.44203

Table S3. LOI and UL-94 vertical burning test results for the uncoated and coated woods.

Samples	UL-94	LOI (%)
W	NR	22.6
G	NR	26.8
G/PAGHR-1	V1	29.6
G/PAGHR-2	V0	33.6
G/PAGHR-4	V0	37.2

Table S4. Thermal properties of the G and G/PAGHR coatings under nitrogen respectively.

Sample	$T_{5\%}$ (°C)	T_{max} (°C)	CY (wt%) （700）
G	219.1	340.1	17.8
G/PAGHR-1	195.4	336.2	33.3
G/PAGHR-2	257.4	319.6	36.8
G/PAGHR-4	244.3	317.0	41.0
PAGHR	264.4	288.2	41.4

Table S5. Cone calorimetry data for the uncoated and coated woods.

Samples	HRR (kW/m^2)	THR (MJ/m^2)
W	193	33
G	309	34
G/PAGHR-1	148	21
G/PAGHR-2	135	17
G/PAGHR-4	77	17
